# Modeling Structure–Activity Relationship of AMPK Activation

**DOI:** 10.3390/molecules26216508

**Published:** 2021-10-28

**Authors:** Jürgen Drewe, Ernst Küsters, Felix Hammann, Matthias Kreuter, Philipp Boss, Verena Schöning

**Affiliations:** 1 Medical Department, Max Zeller Söhne AG, CH-8590 Romanshorn, Switzerland; matthias.kreuter@zellerag.ch; 2 Independent Researcher, D-79427 Eschbach, Germany; kuesterseschbach@aol.com; 3 Clinical Pharmacology and Toxicology, Department of General Internal Medicine, Inselspital University Hospital, CH-3012 Bern, Switzerland; felix.hammann@insel.ch (F.H.); verena.schoening@insel.ch (V.S.); 4 Max Delbrück Center for Molecular Medicine in the Helmholtz Association, D-13125 Berlin, Germany; philipp.boss@posteo.de

**Keywords:** AMPK activator, machine learning, random forest, support vector machine, logistic regression, deep learning, QSAR

## Abstract

The adenosine monophosphate activated protein kinase (AMPK) is critical in the regulation of important cellular functions such as lipid, glucose, and protein metabolism; mitochondrial biogenesis and autophagy; and cellular growth. In many diseases—such as metabolic syndrome, obesity, diabetes, and also cancer—activation of AMPK is beneficial. Therefore, there is growing interest in AMPK activators that act either by direct action on the enzyme itself or by indirect activation of upstream regulators. Many natural compounds have been described that activate AMPK indirectly. These compounds are usually contained in mixtures with a variety of structurally different other compounds, which in turn can also alter the activity of AMPK via one or more pathways. For these compounds, experiments are complicated, since the required pure substances are often not yet isolated and/or therefore not sufficiently available. Therefore, our goal was to develop a screening tool that could handle the profound heterogeneity in activation pathways of the AMPK. Since machine learning algorithms can model complex (unknown) relationships and patterns, some of these methods (random forest, support vector machines, stochastic gradient boosting, logistic regression, and deep neural network) were applied and validated using a database, comprising of 904 activating and 799 neutral or inhibiting compounds identified by extensive PubMed literature search and PubChem Bioassay database. All models showed unexpectedly high classification accuracy in training, but more importantly in predicting the unseen test data. These models are therefore suitable tools for rapid in silico screening of established substances or multicomponent mixtures and can be used to identify compounds of interest for further testing.

## 1. Introduction

The adenosine monophosphate (AMP) activated protein kinase (AMPK) plays a master role in regulating cellular metabolism [1]. Its regulation is critical for many cellular functions, such as lipid, glucose, and protein metabolism; cellular growth; and mitochondrial biogenesis and autophagy [2]. The cellular mode of action suggests a beneficial clinical effect in various metabolic and degenerative diseases, ageing, as well as diabetes, cancer, and viral infection [3].

AMPK adapts cellular metabolism and cell growth to the supply of energy at two levels: At the central level, its hypothalamic activity is regarded as the key negative regulator of sympathetically activated thermogenesis, integrating different peripheral hormonal signals as well as drugs such as thyroid hormone, estrogens, and metabolites with different hypothalamic networks and food signals [4,5,6,7]. In peripheral cells, AMPK senses the loss of cellular energy as an increasing AMP/ATP ratio. AMPK is activated and in turn activates catabolic pathways, improves cellular glucose uptake, and inhibits anabolic reactions [3].

Numerous clinical studies showed the efficacy of metformin—an indirect AMPK activator—in type 2 diabetic patients, resulting in its clinically acceptance as first line initial pharmacologic management of increased blood glucose in adults with type 2 diabetes mellitus (American Diabetes Association [8]). AMPK inhibits the mTOR (mechanistic target of rapamycin) complex 1, a nutrient sensitive master regulator of cell growth, angiogenesis, and metabolism that is activated by growth factors especially in tumors [9]. Several in vitro studies with the AMPK activator metformin showed the induction of cell cycle arrest in different tumor models (among others in breast cancer [10], and prostate cancer [11]). Therefore, the indirect activator metformin has been investigated in many clinical studies in patients with different types of cancer [12].

For a variety of different compounds, direct and/or indirect AMPK activation has been described: direct activators bind to the catalytic α subunit of AMPK allowing it to be phosphorylated at Thr172 or to the regulatory γ-subunit for allosteric activation [1]. Physiological direct activators are AMP, to a smaller extent ADP; pharmacological direct activators comprise among others AICAR (5-aminoimidazole-4-carboxamide ribose), A-769662 and salicylate and a variety of newly synthesized compounds.

Indirect activation is controlled by upstream kinases (such as liver kinase B1 (LKB1), calcium/calmodulin-dependent protein kinase kinase (CaMKKβ), or transforming growth factor-β (TGFβ-activated kinase 1 (TAK1))), which phosphorylate Thr172 of the α-subunit [1,13]. In general, a multitude of compounds may indirectly activate AMPK by virtue of diverse mechanisms, which lower cellular levels of ATP. Examples are drugs, such as metformin, and also a variety of natural compounds [14]—in particular, constituents of *Cimicifuga racemosa* (black cohosh) [15].

These natural compounds are usually contained in mixtures of a large number of structurally different chemical compounds which can directly or indirectly activate AMPK. Therefore, our goal was to develop a screening tool that could handle the heterogeneity of different activation pathways complementing in vitro assays that provide information on direct influence on the enzyme. 

Frequently, (quantitative) structure–activity analyses are performed to predict the pharmacological or toxicological activity of new compounds. Nowadays, this is often done in vitro in extensive high-throughput experiments, where the desired pharmacological effects are measured in specialized cell culture assay systems. Molecular modeling of the target structure itself has also been used to investigate the structural requirements for binding to the target. However, machine learning-based QSAR methods are also able to more quickly and with less effort linking structural information to more general questions: e.g., an effect caused by different mechanisms, such as AMPK activation, but also more complex effects such as toxic adverse drug reactions [16,17,18,19,20], drug–drug interactions [21,22] or to predict clinical therapeutic effect. Last but not least, these machine learning methods could be used to screen on a large-scale drugs approved for a different indication for new indications in a repurposing approach [23].

Since machine learning algorithms can model complex (unknown) relationships and patterns, we used these methods to model the structural requirements for the pharmacological effect of AMPK activation. 

## 2. Results

**Figure 1:**
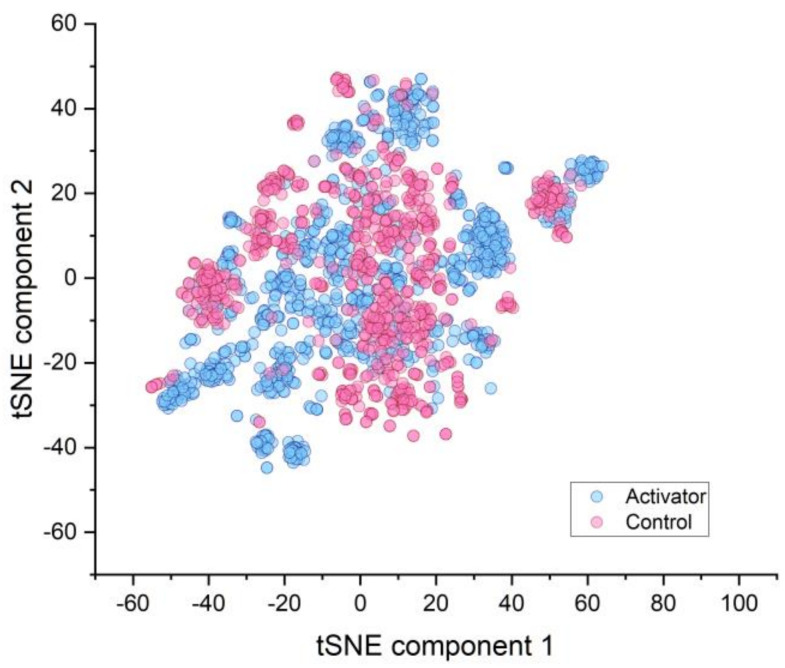
*t*-distributed stochastic neighbor embedding (tSNE) analysis: AMPK activators and controls.

The activators of AMPK and controls (either no activation or inhibition of AMPK) were compared by the *t*-distributed stochastic neighbor embedding (tSNE) analysis to graphically assess the applicability domain of the database compounds (Figure 1). 

**Figure 2:**
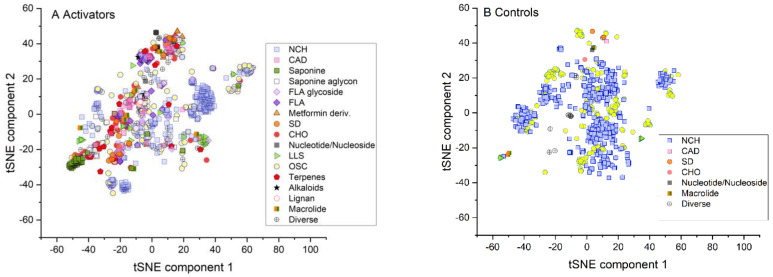
tSNE analysis of chemically classified activators and controls separated by chemical structure. (**A**) AMPK activators (N = 904); (**B**) AMPK control (N = 799).

In this comparison, the controls appeared to be mainly distributed in the center of the plot, while activators were preferentially distributed in the periphery, indicating different clusters (Figure 1). The chemical characterization of activators and inhibitors was done according to suggestions by Sharma and Kumar [14], who described groups of chemical compounds that were frequently associated with AMPK activation in the literature (Figure 2). The visualization of these proposed chemical groups showed clear qualitative differences between the activators and the controls.

### 2.1. Similarity of Groups

The mean standardized distances (mean; standard deviation) was higher (45.8; 18.8) within the activators than within the controls (40.2; 22.2), indicating a higher heterogeneity in the former group. This reflects the most diverse activation pathways of the AMPK. 

### 2.2. Statistical Comparison of Datasets

As described in the method section, for each database instance (compound), 1445 chemical descriptors (PaDEL) have been calculated. All descriptors (features) have been compared between controls and activators by unpaired *t*-tests assuming unequal variances. From all 1445 features, 835 were significantly different between the two groups. Among others, selection of intuitive features is reported in Table 1. Compared to the controls, the activators had on average more acidic groups (*p* < 0.001), more aromatic atoms and aromatic bonds (both *p* < 0.001) and a higher number of nitrogen atoms (*p* < 0.02). In addition, they had a significantly (*p* < 0.001) smaller number of 5- to 7-membered rings containing heteroatoms (N, O, P, S, or halogens).

Finally, activators had a significantly smaller molecular weight (*p* < 0.001) and a higher lipophilicity (*p* < 0.001). Both, controls and activators had on average less than one failure of the Lipinski’s rule of five, which qualify them as drug-like compounds. 

**Figure 3:**
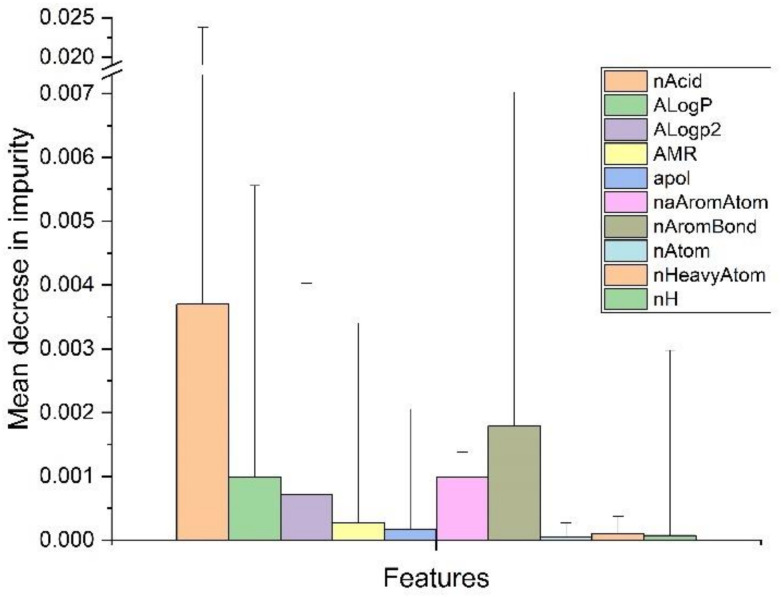
Feature importance (standard deviation) of the first 10 features for random forest classification; nAcid = number of acidic groups; ALogP = Ghose-Crippen LogKow; ALogP2 = square of ALogP; AMR = molar refractivity; apol = sum of the atomic polarizabilities (including implicit hydrogens); naAromAtom = number of aromatic atoms; nAromBond = number of aromatic bonds; nAtom = number of atoms; nHeavyAtom = number of heavy atoms; nH = number of hydrogen atoms.

There were also large differences between features in classification, as demonstrated, for example, in the random forest classification (Figure 3).

### 2.3. Random Forest Classification (RFC)

The following hyperparameter settings were found to be optimal by grid-search analysis with a mean accuracy of 91.6% on the training set: using the Gini impurity criterion; determined minimal samples split = 6; minimal samples leaf = 2; the number of estimators (created trees) was set to 100. The bootstrap option was set to “False”. Finally, the maximum number of features was determined by the square root of the number of features. For all other settings, default values were used, including automatic calculation of weights to account for different class sizes. 

### 2.4. Support Vector Machine Classification (SVM-C)

The following hyperparameter settings were found to be optimal by grid-search analysis with a mean accuracy of 91.0% on the trainings set: the regularization parameter C = 10. The strength of the regularization is inversely proportional to C. This value is strictly positive. The penalty is a squared l2 penalty. The radial basis function (RBF) kernel was chosen with kernel coefficient gamma = “scale”. That means that the program uses 1/(n_features × X.var()) as value of gamma. Finally, the class weight was set to “balanced” to include automatic calculation of weights to account for different class sizes. 

### 2.5. Stochastic Gradient Boosting (SGB) Analysis

The following hyperparameter settings were found to be optimal by grid-search analysis with a mean accuracy of 99.3%: the optimal loss function was “exponential” that recovers the AdaBoost algorithm. The optimal learning rate was 0.1, maximum dept was 3, the number of estimators 1000 and the fraction of samples to be used for fitting the individual base learners was 0.7. Furthermore, the class weight was set to “balanced” to include automatic calculation of weights to account for different class sizes. 

### 2.6. Logistic Regression Classification (LRC)

Different optimizers (“Newton- cg” (Newton conjugate gradient optimizer), “lbfgs” (limited memory Broyden-Fletcher-Goldfarb-Shanno optimizer)—a quasi-Newton method and “liblinear”—a linear classifier for smaller datasets) have been tested. The following hyperparameter settings were found to be optimal by grid-search analysis: the regularization parameter C was set to 0.1; for regularization, the penalty by the l_2_-norm and as optimizer the Newton-cg was applied. The class weight was “balanced” to include automatic calculation of weights to account for different class sizes. 

### 2.7. Deep Neural Network (DNN) Analysis 

As the last approach, a deep neural network analysis was performed using tensorflow and its API keras. The following sequential model approach was applied: The optimum hyperparameter were: Adams optimizer with learning rate = 0.001, batch size = 128, and 50 learning epochs. The input layer used 1000 units (neurons), three hidden layers with neurons, and the activation function Elu. The code of the model is given in Best_DNN_model.pdf in https://github.com/cptbern/QSAR_AMPK, accessed on 27 October 2021. 

### 2.8. Test Performance

**Figure 4:**
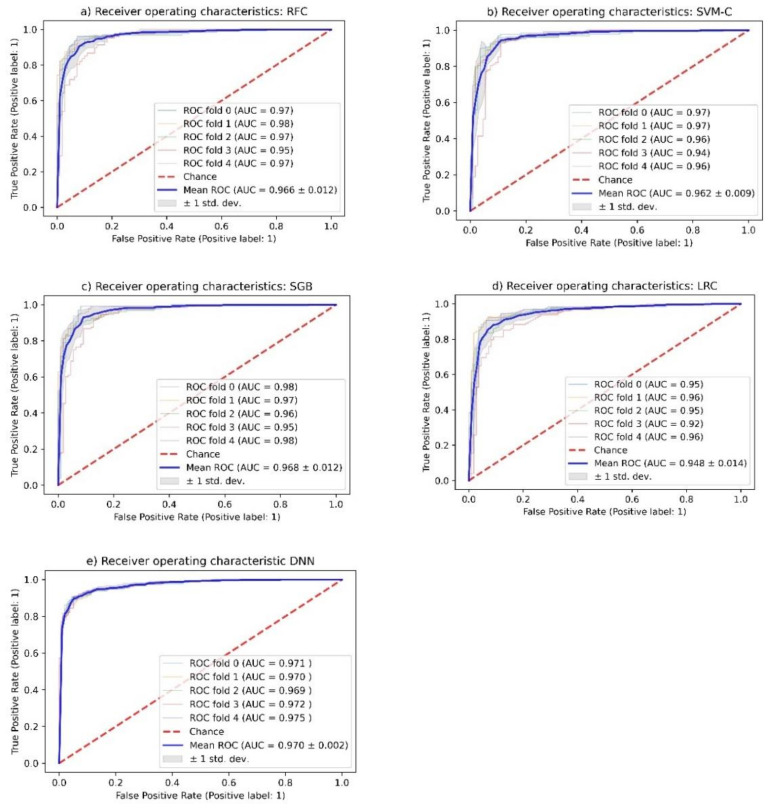
Receiver operating characteristic (ROC) of the investigated methods. (**a**) Random Forest classifier, (**b**) Support Vector Machine classifier, (**c**) Stochastic Gradient Boosting classifier, (**d**) Logistic Regression classifier, and (**e**) Deep Neural Network classifier.

The performance of these models is given in Table 1. The ROC-curves for all models are given in Figure 4. 

All investigated models showed an unexpectedly high accuracy in the training but more importantly in the unseen test data set. The accuracy of all models was comparable between 91.0% and 93.0%. No clear advantage of one method over the others could be identified.

To rule out overfitting of the data, Y-randomization of the response variables was applied, achieving only about 52% accuracy for all methods (an indication that final models have picked up on actual patterns rather than statistical noise). In addition, a 5-fold cross-validation was performed, which confirmed the good predictivity. ROC curves confirmed the high accuracy of the models.

## 3. Discussion

All models showed very good performance in discriminating AMPK activators from controls. The 5-fold cross-validation and the Y-randomization of the response variable makes it unlikely that the high accuracy of all methods used is due to overfitting of the data or to chance.

However, the DNN model appeared to have lower variability compared to the others (Figure 4). It can be speculated that deep learning networks cope better with complex, heterogeneous datasets than the other machine learning methods. 

It is obvious that activation of AMPK is obtained via different (direct and indirect) mechanisms (Figure 4) explaining the structural heterogeneity (Figure 2). In order to achieve comprehensive prediction, it is therefore also necessary to have a sufficient number of different activators in the database acting through any of these mechanisms. 

Although we had an excellent prediction of our unseen test data set, we could not exclude missing mechanisms. Furthermore, we have to consider that machine learning modeling always carries the limitation that classification provides only probabilities which require verification by direct in vitro or in vivo experiments. However, these experiments are time- and resource-consuming. Therefore, one goal of machine learning prediction is to facilitate the selection of suitable candidates from a range of possible candidates.

In the literature (PubMed search with terms “AMPK” and “QSAR”), several QSAR models for prediction of AMPK activation have been published so far [24,25,26,27,28,29,30]. However, all of these models used pharmacophore docking analyses or homology models or structure-, ligand-, or fragment-based design and focused exclusively on direct AMPK activators. In contrast to these approaches, as far as we know, we were the first to explicitly extend our models by including all compounds that showed evidence of AMPK activation, regardless of the activation pathway, whether direct or indirect. Therefore, our approach is more general and better accounts for the well-described heterogeneity of AMPK activation (Figure 4). Finally, it is easily adaptable to unseen data. However, there is also a limitation of our approach, because it does not provide information about the activation pathway and the type of activation (direct or indirect). 

Only a smaller fraction of activators interacts directly with AMPK by binding to specific activating or allosteric binding sites of the enzyme. The majority of activators act indirectly. They bind to upstream regulatory sites that, when activated, in turn phosphorylate and activate AMPK. This places structural requirements on these activators to bind to these sites, contributing to the structural diversity of these compounds (Figure 2). 

**Figure 5:**
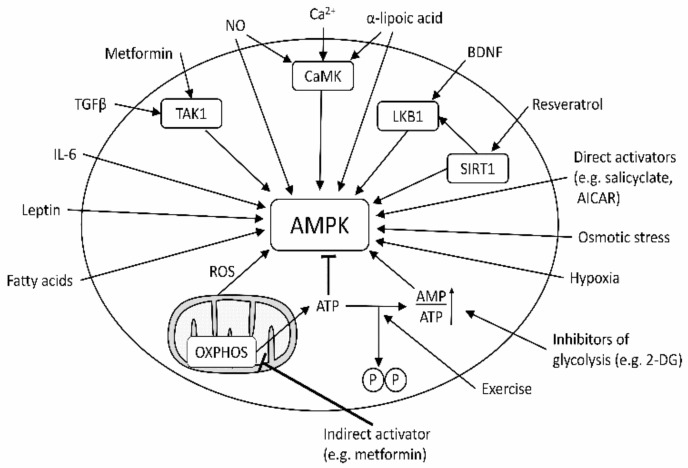
Activation pathways of AMPK.

AMPK is an important enzyme sensing and controlling energy supply and different cellular functions—e.g., carbohydrate cellular entry and metabolism, reactive oxygen species (ROS) generation, apoptosis, cellular growth, and mitochondrial biogenesis and autophagy [1]. Since the enzyme is at the intersection of several important cellular pathways, it is obvious to assume the existence of different modes of activation (Figure 5).

Machine learning methods for AMPK activation are important: the relevance of AMPK in the pathogenesis of different diseases and their treatment was discussed above and new and old treatment modalities will be assessed with regard to their potential to modulate AMPK activity in the sense of repurposing of established (herbal) drugs.

To investigate herbal drugs, experiments are complicated by the fact that extracts are multi-substance mixtures and the required pure substances are often not sufficiently isolated and/or therefore not sufficiently available. Therefore, our extended method offers attractive new applications in the extended screening of these multi-substance mixtures to identify lead substances of interest for further intensified testing.

## 4. Materials and Methods

### 4.1. Data

The AMPK data set was based on extensive literature search of AMPK activators and inhibitors that was performed in PubMed (https://pubmed.ncbi.nlm.nih.gov/, accessed on 27 October 2021) using search terms:

- “AMPK AND activation”
- “AMPK AND inhibition”

In addition, the Bioassay database of PubChem Substance and Compound databases (https://pubchem.ncbi.nlm.nih.gov/, accessed on 27 October 2021) was used when EC_50_ was ≤0.1 μM to identify proven activators. In addition, compounds were included that were confirmed activators by at least one PubMed-listed publication. On the other hand, tested compounds shown to be inactive for AMPK activation or showing inhibitory function or compounds described in the literature as inhibitors of AMPK formed the control group for this analysis. 

### 4.2. Data Preprocessing

Chemical structures were coded by the Simplified Molecular-Input Line-Entry System (SMILES; isomeric if available, canonical otherwise) that were taken directly from PubChem or when no PubChem ID was available were determined by MarvinSketch version 19.22 (ChemAxon). SDF Files were generated by Open Babel (version 2.21) https://openbabel.org, accessed on 27 October 2021. These files were used to calculate physicochemical descriptors with PaDEL descriptor software http://www.yapcwsoft.com/dd/padeldescriptor/, accessed on 27 October 2021. We computed the entire range of available 1D and 2D descriptors [31] for all compounds. 

Data preprocessing (curation) involved removing any double entries, salts and mixtures, and proteins from SMILES structures and focused on small molecular weight drug-like compounds. Tautomers were not standardized. Compounds and/or descriptors with empty descriptor values were excluded from analysis yielding N = 904 and N = 799 valid cases for activators and controls, respectively and N = 1445 features (descriptors). A complete list of used activators and controls is given:

- with their names, smiles codes, PubChem IDs, and PubMed IDs (Compounds.csv); and
- with all calculated PaDel descriptors (Data.csv) in https://github.com/cptbern/QSAR_AMPK, accessed on 27 October 2021.

### 4.3. Validation

Validation of models were based on OECD Principles for (Q)SAR Validation [32]. Validation was based on the random split of data into training and test data. From the full data set (N = 1703), 70% were randomly chosen for training (N = 1192) and 30% for the final test data set (N = 511), respectively. From the training data, 80% (N = 953) of the data were randomly chosen as validation training dataset and 20% (N = 239) as the validation test dataset. Following internal validation, the final model was used on the full training data set (N = 1192) to predict the previously unseen instances of the test data set (30% of activators (N = 262) and controls (N = 249), each) as external validation.

Training data were used to optimize model hyperparameters and train the models. After training hyperparameters were optimized on the training data set, final model learning was performed using 5-fold internal cross-validation. Furthermore, the training was repeated after randomization (N = 100) of the response variable (*Y-randomization*) as additional validation [33].

### 4.4. Similarity

Similarity or heterogeneity within the activators and controls was calculated in each case by mean Euclidean distance between each element to all other elements of the group based on the standardized values of the descriptors

(1) $$\mathrm{Mean}\;\mathrm{standardised}\;\mathrm{distance}=\frac{\sqrt{\sum_{i,j}{\left(x_{i}-x_{j}\right)}^{2}}}{S}$$

where, x_i_ and x_j_ denote the *i*-th and *j*-th instance and each descriptor values of the respective group, respectively (i, j = 0, …, N, the number of elements of the group, i ≠ j), S denotes the number of pairwise differences. 

For classification of data, different machine learning algorithms from the open scikit-learn python-based machine learning framework (https://scikit-learn.org/stable/, accessed on 27 October 2021) were applied: tuning of hyperparameters was done by grid search estimation (sklearn *GridSearchCV*) where relevant.

### 4.5. Machine Learning Models

All calculations were performed using Python 3.9.1 (https://www.python.org/, accessed on 27 October 2021). Graphical analysis was done with OriginPro, Version 2021. OriginLab Corporation, Northampton, MA, USA and Matplotlib, version 3.3.3 (https://matplotlib.org/, accessed on 27 October 2021). 

The structural relationship of the high dimensional data of activators, controls and random samples were visualized by non-linear embedding into a two-dimensional space by the *t*-distributed stochastic neighbor embedding technique (*tSNE*) to visualize the applicability domain of the database [34]. In order to better visualize the structural diversity, activators and controls were chemically classified similar to the suggestions of Sharma and Kumar [14] as alkaloids; cinnamic acid derivatives (CAD); carbohydrates (CHO); flavonoid derivatives (FLA); lignans, lipid-like structures (LLS: ≥ C8-chain); macrolides; metformin derivatives; nitrogen-containing heterocycles (NCH); nucleotide/nucleoside derivatives; organic sulfur-containing structures (OSC); saponins and their aglycons; sugar derivatives (SD); stilbenes and terpenes.

### 4.6. Random Forest Classification (RFC) 

Random forest is an ensemble method [35]. These methods combine several base estimators in order to improve generalizability and robustness compared to single estimators (decision trees). A sequence of base estimators is built and each of these estimators tries to reduce the bias of the combined estimator. Random forests are a powerful decision tree algorithm for classification. Hyperparameters were tuned by grid search analysis on number of estimators, maximum features used, maximum depth of trees, minimum samples split, minimum samples leaf, and impurity criterion. No bootstrap sampling was performed.

### 4.7. Stochastic Gradient Boosting Classification (SGB) 

Stochastic gradient boosting (SGB) classification [36] also belongs to the ensemble methods. Hyperparameters were tuned by grid search analysis on number of estimators, maximum depth of trees, the loss function (deviance (=logistic regression) or exponential (=AdaBoost algorithm)), the learning rate, and the fraction of samples to be used for fitting the individual base learners.

### 4.8. Support Vector Machine Classification (SVM-C) 

Support Vector Machine Classification [37] tries to separate the two classes in the n-dimensional feature space by constructing a (n-1)-dimensional hyperplane that maximizes the margin between the two classes. Most important is the choice of the best kernel, a function to transform the data to a higher-dimensional space: Here, grid search evaluated the radial basis function (RBF, Gaussian kernel), the sigmoid and the polynomial kernels. Furthermore, the regularization parameter C and the best the kernel coefficient gamma were estimated. 

### 4.9. Logistic Regression Classification (LRC) 

Logistic Regression Classification [38] is used to estimate the probability $\hat{p}$ that an instance belongs to a class

(2) $$\hat{p}={\;h}_{\theta }\left(x\right)=\sigma \left(\theta^{T}\;\cdot \;x\right),$$

using the logistic function

(3) $$\sigma \left(t\right)=\frac{1}{1+{\;e}^{-t}}.$$

Classification for two classes denotes with 0 and 1 will be obtained

(4) $$\hat{y}=\;\sigma \left(t\right)=\left\{\begin{array}{c} 0,\;\;\hat{p}<0.5\\ 1,\;\;\hat{p}\geq 0.5 \end{array}\right.$$

Different solvers (Newton-cg, lbfgs and liblinear), the regularization parameter C and the penalty (l_1_-norm, l_2_-norm, and elastic net) were evaluated.

### 4.10. Deep Learning Neural Network (DNN)

As last approach, a deep neural network analysis was performed using tensorflow, version 2.3.1 (https://www.tensorflow.org/, accessed on 27 October 2021) and its associated neural networking API tensorflow.keras (https://www.tensorflow.org/api_docs/python/tf/keras, accessed on 27 October 2021). The following sequential model approach was applied: an input layer with 1000 units and “elu” activation and a variable number of hidden dense layers with “elu” activation and finally a dense output layer with sigmoid activation was used: The input layer was connected to one to three hidden dense layer and these to an output layer giving the prediction of the model. An extensive grid search was performed to estimate the unknown parameters (n_hidden: number of hidden dense layers: 0, 1, 2, 3 for the number of hidden dense and dropout layers; number of neurons: 750, 500, and 250 units for the hidden dense layers; the Adams or RMSprop optimizers; 0.0001 to 5 for the learning rates of the optimizer and 16 to 1024 for the batch sizes). As kernel initializer the HeNormal, and as bias initializer Constant(value = 0) were used. Number of features (descriptors) was 1445 and the number of targets was 1. This search was done using the tensorflow scikit learn wrapper and the GridSearchCV module from scikit learn with 5-fold cross-validation. As activation functions the exponential linear unit [ELU(z) = z, for z > 0 and alpha × (exp(x) − 1) if x < 0] and sigmoid [σ(z) = 1/(1 + exp(−z))] functions were used for hidden and output layers, respectively and as loss function the binary cross-entropy [39] was used.

### 4.11. Model Evaluation

To account for differences in class sizes between activators and controls the ‘balanced’ option was chosen, if available, to perform automatic calculation of class weights. Data were divided into disjunct parts using the *sklearn.model_selection preprocessing* procedure *train_test_split* using a test_size of 30% and 70% for training. A 5-fold cross-validation (CV) was applied. 

Activators and controls populations were compared with 100,000 random samples drawn from PubChem database using PubChemPy tools that provide a way to interact with PubChem in Python (https://pubchempy.readthedocs.io/en/latest/#, accessed on 27 October 2021). The data distributions were compared by *t*-distributed stochastic neighbor embedding analysis using the *sklearn.manifold.TSNE* procedure. This method visualizes high-dimensional data by a two-dimensional representation to graphically assess the applicability domains. Goodness of machine learning modeling is reported as

(5) $$\mathrm{accuracy}=\frac{\mathrm{TP}+\;\mathrm{TN}}{\mathrm{TP}\;+\;\mathrm{TN}\;+\;\mathrm{FP}\;+\;\mathrm{FN}}$$

(6) $$\mathrm{precision}=\frac{\mathrm{TP}}{\mathrm{TP}\;+\;\mathrm{FP}}$$

(7) $$\mathrm{sensitivity}=\frac{\mathrm{TP}}{\mathrm{TP}\;+\;\mathrm{FN}}$$

(8) $$\mathrm{specificity}=\frac{\mathrm{TN}}{\mathrm{TN}\;+\;\mathrm{FP}}$$

where TP = true positive (activator correctly predicted); FP = false positive (activator incorrectly predicted); TN = true negative (control correctly predicted); FN = (false negative control incorrectly predicted).

Validation was performed as 5-fold cross-validation using *sklearn.KFold* procedure. To evaluate binary classifier output independently of thresholds the receiver operating characteristic (ROC) and its area under the curve (AUC) scores [40] were calculated that assesses the tradeoff between sensitivity by plotting sensitivity versus (1 − specificity). 

## Figures and Tables

**Table 1:** Summary of results of classification of different machine learning methods.

Method	Training Accuracy (%)	Test Accuracy (%)	Y-Randomization ** (%)	Test Precision (%)	Sensitivity (%)	Specificity (%)	AUC *
RFC	91.6	92.6	52.7 ± 2.3	90.3	91.2	94.0	0.968 ± 0.013
SVM-C	91.0	93.0	53.2 ± 2.2	90.1	93.5	92.4	0.962 ± 0.009
SGB	91.3	93.0	52.8 ± 2.2	90.7	92.0	94.0	0.968 ± 0.012
LRC	90.8	91.0	52.6 ± 2.1	89.2	97.4	94.8	0.948 ± 0.014
DNN	91.6	90.6	53.0 ± 1.8	87.6	90.2	91.1	0.970 ± 0.002

Test set (number): activator (262), control (249); * AUC = Area under the receiver operating characteristics curve. ** N = 100 permutations.

## Data Availability

Complete list of used activators and controls are given: with their names, smiles codes, PubChem IDs, and PubMed IDs (Compounds.csv) and with all calculated PaDel descriptors (Data.csv) and source codes of all models in https://github.com/cptbern/QSAR_AMPK, accessed on 27 October 2021.

## Sample Availability

Samples of the compounds not available rom the authors, however, names and descriptors are given at https://github.com/cptbern/QSAR_AMPK, accessed on 27 October 2021.
